# Supplementary material for: NF-ULA: Normalizing flow-based unadjusted Langevin algorithm for imaging inverse problems
Source: SIAM J Imaging Sci. Author manuscript; Available in PMC 2026 Oct 16. (PMC7619580; doi:10.1137/23m1581807)
Supplement: Supplementary file 1 [file EMS218492-supplement-Appendix.pdf]

Appendix A. Proofs.

A.1. Proof of Lemma 3.1.

Proof. For a constant $R_0 > 0$, let

$$B(0, R_0) := \left\{ z \in \mathbb{R}^d : \|z\|_2 \leq R_0 \right\}$$

be the closed ball of radius R_0 centered at the origin. Since $C \subset \mathbb{R}^d$ is compact, there exists $R_0 > 0$ such that $C \subset B(0, R_0)$. Therefore, for all $x \notin B(0, R_0)$, it follows that

$$\|x - \Pi_C(x)\|_2 \overset{\text{(a)}}{\geqslant} \left\|x - \Pi_{B(0,R_0)}(x)\right\|_2 \overset{\text{(b)}}{\geqslant} \|x\|_2 - R_0 \geq 0,$$

where (a) is true since $C \subset B(0, R_0)$ and (b) follows from the triangle inequality. Then, for all $k \in \mathbb{N}$, the following holds:

$$\begin{aligned}
&\int_{\mathbb{R}^d \setminus B(0,R_0)} \|x\|^k \exp\left(-\frac{\|x - \Pi_C(x)\|_2^2}{2\lambda}\right) \mathrm{d}x \\
&\leqslant \int_{\mathbb{R}^d \setminus B(0,R_0)} \|x\|^k \exp\left(-\frac{(\|x\|_2 - R_0)^2}{2\lambda}\right) \mathrm{d}x \\
&\leqslant \int_{\mathbb{R}^d \setminus B(0,R_0)} \|x\|^k \exp\left(-\frac{\|x\|_2^2 - 2R_0^2}{4\lambda}\right) \mathrm{d}x \\
&< +\infty,
\end{aligned}$$

where the last inequality follows from the fact that k-order moments of Gaussian distribution are finite for any k. ∎

A.2. Proof of Proposition 3.3.

Proof. Without loss of generality, we only need to consider the cases when the total number of layers is $k = 1, 2$.

(1) We firstly consider the case that $k = 1$ and $T^{-1} = G$ is a composition of only a one-layer coupling network. Then (3.4) can be simplified as:

$$G_j(x_j, x_{<j}) = \varphi_j(x_{<j})x_j + \eta_j(x_{<j}),\ j = 1, \cdots, d. \tag{A.1}$$

Since $\forall r < j$, G_r is independent of x_j and the diagonal of the Jacobian is $(J_G(x))_{j,j} = \varphi_j(x_{<j})$, from the change of variables

$$\begin{aligned} q(x) &= q_z(z) \left|\det J_T(z)\right|^{-1} \\ &= q_z\left(T^{-1}(x)\right) \left|\det J_{T^{-1}}(x)\right|, \end{aligned} \tag{A.2}$$

we have that

$$\begin{aligned} \log q_\theta(x) &= \log q_z\left(G(x)\right) + \log\left|\det J_G(x)\right| \\ &= -\frac{1}{2}\left\|G(x)\right\|_2^2 + \log\left|\det J_G(x)\right| + \text{const.} \\ &= -\frac{1}{2}\left\|G(x)\right\|_2^2 + \sum_{j=1}^{d} \log\left|\varphi_j(x_{<j})\right| + \text{const.} \\ &\leqslant \sum_{j=1}^{d} \log\left|\varphi_j(x_{<j})\right| + \text{const.} \end{aligned}$$

Since φ_j is a bounded function $\forall j$, it follows that $\log|\varphi_j(x_{<j})|$ is upper bounded for all j and $\log q_\theta(x)$ is upper bounded on $\mathbb{R}^d$.

(2) Secondly, assume that $k = 2$ and $T^{-1} = G \circ H(x)$, where $H : x \mapsto \omega$ and $G : \omega \mapsto z$. Similarly, we have that

$$\begin{aligned} \log q_\theta(x) &= \log q_z\left(G \circ H(x)\right) + \log\left|\det J_{G\circ H}(x)\right| \\ &= -\frac{1}{2}\left\|G \circ H(x)\right\|_2^2 + \log\left|\det J_G(\omega)\right| + \log\left|\det J_H(x)\right| + \text{const.} \\ &= -\frac{1}{2}\left\|G \circ H(x)\right\|_2^2 + \sum_{j=1}^{d}\left(\log\left|\varphi_j^{(2)}(\omega_{<j})\right| + \log\left|\varphi_j^{(1)}(x_{<j})\right|\right) + \text{const.} \\ &\leqslant \sum_{j=1}^{d}\left(\log\left|\varphi_j^{(2)}(\omega_{<j})\right| + \log\left|\varphi_j^{(1)}(x_{<j})\right|\right) + \text{const.} \end{aligned}$$

Since $\varphi_j^{(1)}$ and $\varphi_j^{(2)}$ are bounded functions $\forall j$, it follows that $\log\left|\varphi_j^{(2)}(\omega_{<j})\right| + \log\left|\varphi_j^{(1)}(x_{<j})\right|$ is upper bounded for all j and $\log q_\theta(x)$ is upper bounded on $\mathbb{R}^d$. ∎

A.3. Proof of Proposition 3.5.

Proof. By Assumption 3.2, we have that

$$\int_{\mathbb{R}^d} (1+\Phi_1(\tilde{x})) \exp\left[c_0\Phi_1(\tilde{x}) - \iota_C^{(\lambda)}(\tilde{x})\right] q_\theta^\alpha(\tilde{x})\mathrm{d}\tilde{x} < +\infty,$$

and we conclude the proof from Proposition 2.3 of [56]. ■

A.4. Proof of Lemma 3.7.

Lemma A.1. *Let Assumption 3.6 be true. Then, $\nabla \log p_\lambda(x|y)$ is Lipschitz continuous if and only if $\nabla \log q_\theta(x)$ is Lipschitz continuous.*

Proof. Since Assumption 3.6 is satisfied, from Algorithm 2.1 and (2.13) we have that $\nabla \log p_\lambda(x|y)$ is Lipschitz continuous if and only if $\alpha\nabla \log q_\theta(x) + (\Pi_C(x) - x)/\lambda$ is Lipschitz continuous.

From Proposition 12.28 in [9], the operator $(\mathrm{Id} - \mathrm{Prox}_{\iota_C})$ is firmly non-expansive, i.e., for all $x, y \in \mathbb{R}^d$,

$$\begin{aligned}\|(\Pi_C(x) - x) - (\Pi_C(y) - y)\|_2^2 &\leqslant \langle(\Pi_C(x) - x) - (\Pi_C(y) - y), x - y\rangle \\ &\leqslant \|(\Pi_C(x) - x) - (\Pi_C(y) - y)\|_2 \|x - y\|_2 .\end{aligned}$$

Therefore, $(\Pi_C(x) - x)/\lambda$ is $1/\lambda$-Lipschitz. Hence, for any $\alpha > 0$, $\nabla \log p_\lambda(x|y)$ is Lipschitz-continuous if and only if $\nabla \log q_\theta(x)$ is Lipschitz-continuous. ■

A.5. Proof of Proposition 3.9.

Proof. Without loss of generality, we only need to consider the cases when the total number of layers is $k = 1, 2$.

(1) We firstly consider the case that $k = 1$ and $T^{-1} = G$ is a composition of only a one-layer coupling network. Then (3.5) can be simplified as:

$$G_j(x_j, x_{<j}) = \varphi_j(x_{<j})x_j + \eta_j(x_{<j}),\ j = 1, \cdots, d. \tag{A.3}$$

Since $\forall r < j$, G_r is independent of x_j and the diagonal of the Jacobian is $(J_G(x))_{j,j} = \varphi_j(x_{<j})$, from the change of variables

$$\begin{aligned} q(x) &= q_z(z)\left|\det J_T(z)\right|^{-1} \\ &= q_z\left(T^{-1}(x)\right)\left|\det J_{T^{-1}}(x)\right|, \end{aligned} \tag{A.4}$$

we have that

$$\begin{aligned}\log q_\theta(x) &= \log q_z\left(G(x)\right) + \log\left|\det J_G(x)\right| \\ &= -\frac{1}{2}\|G(x)\|_2^2 + \log\left|\det J_G(x)\right| + \mathrm{const.} \\ &= -\frac{1}{2}\|G(x)\|_2^2 + \sum_{j=1}^d \log\left|\varphi_j(x_{<j})\right| + \mathrm{const.}\end{aligned}$$

Taking the gradient of both sides w.r.t. x, we get

$$\nabla \log q_\theta(x) = -\left(J_G(x)\right)^T G(x) + \sum_{j=1}^{d} \nabla \log \varphi_j(x_{<j}). \tag{A.5}$$

Since φ_j is a constant function, we have that $\nabla \log \varphi_j = 0$. Furthermore as η_j is Lipschitz and $\forall r < j$, $\frac{\partial \eta_j}{\partial x_r}$ is piecewise constant on $\mathbb{R}$, $\frac{\partial \eta_j}{\partial x_r}$ is hence bounded. Meanwhile, $(J_G(x))_{j,r} = \frac{\partial \eta_j}{\partial x_r}$, therefore every element of $J_G(x)$ is a bounded piecewise constant function of x. Then both $G(x)$ and $(J_G(x))^\top G(x)$ are Lipschitz, therefore $\nabla \log q_\theta(x)$ is Lipschitz.

(2) Secondly, assume that $k = 2$ and $T^{-1} = G \circ H(x)$, where $H : x \mapsto \omega$ and $G : \omega \mapsto z$. Similarly, we have that

$$\begin{aligned}
\log q_\theta(x) &= \log q_z\left(G \circ H(x)\right) + \log |\det J_{G\circ H}(x)| \\
&= -\frac{1}{2}\|G \circ H(x)\|_2^2 + \log |\det J_G(\omega)| + \log |\det J_H(x)| + \text{const.} \\
&= -\frac{1}{2}\|G \circ H(x)\|_2^2 + \sum_{j=1}^{d}\left(\log\left|\varphi_j^{(2)}(\omega_{<j})\right| + \log\left|\varphi_j^{(1)}(x_{<j})\right|\right) + \text{const.}
\end{aligned}$$

and

$$\begin{aligned}
\nabla \log q_\theta(x) &= -\left(J_{G\circ H}(x)\right)^T G \circ H(x) + 0 \\
&= -\left(J_G(H(x)) J_H(x)\right)^T G \circ H(x).
\end{aligned} \tag{A.6}$$

Since every element of $J_H(x)$ is a bounded piecewise constant function of x, every element of $J_G(w)$ is a bounded piecewise constant function of w, and meanwhile $w = H(x)$ is continuous w.r.t. x, then every element of $J_{G\circ H}(x)$ is a bounded piecewise constant function of x. Then both $G \circ H(x)$ and $(J_{G\circ H}(x))^\top G \circ H(x)$ are Lipschitz, therefore $\nabla \log q_\theta(x)$ is Lipschitz. ∎

A.6. Proof of theorem 3.11.

Proof. Denote $R_{\mathrm{C}} = \sup\{\|x_1 - x_2\| : x_1, x_2 \in \mathrm{C}\}$. Since we have $2\lambda(\alpha\mathrm{L} - \mathrm{m}_y) \leqslant 1$, from A 3.8, A 3.10, $b_\lambda(x)$ in (3.6) and the Cauchy-Schwarz inequality we have that for any $x_1, x_2 \in \mathbb{R}^d$,

$$\begin{aligned}
\langle b_\lambda(x_1) - b_\lambda(x_2), x_1 - x_2\rangle &\leqslant (-\mathrm{m}_y + \alpha\mathrm{L})\|x_1 - x_2\|^2 - \frac{\|x_1 - x_2\|^2}{\lambda} + \frac{R_{\mathrm{C}}\|x_1 - x_2\|}{\lambda} \\
&\leqslant -\frac{\|x_1 - x_2\|^2}{2\lambda} + \frac{R_{\mathrm{C}}\|x_1 - x_2\|}{\lambda}.
\end{aligned} \tag{A.7}$$

For any $x_1, x_2 \in \mathbb{R}^d$ satisfying $\|x_1 - x_2\| \geqslant 4R_{\mathrm{C}}$, we obtain the contractivity at infinity condition on the drift b_λ

$$\langle b_\lambda(x_1) - b_\lambda(x_2), x_1 - x_2\rangle \leqslant -\frac{\|x_1 - x_2\|^2}{4\lambda}, \tag{A.8}$$

which indicates the strongly convexity at infinity.

After simple computation by letting $x_2=0$ in (A.7), we also have that for any $x \in \mathbb{R}^d$,

$$\langle b_\lambda(x), x\rangle \leqslant -\|x\|^2/(4\lambda) + \sup_{\tilde{x}\in\mathbb{R}^d}\left\{(R_{\mathrm{C}}/\lambda + \|b_\lambda(0)\|)\|\tilde{x}\| - \|\tilde{x}\|^2/(4\lambda)\right\}. \tag{A.9}$$

From A 3.6, A 3.8, $b_\lambda(x)$ in (3.6) and that $(\mathrm{Id}-\Pi_C)/\lambda$ is $1/\lambda$-Lipschitz, we have that for any $x_1, x_2 \in \mathbb{R}^d$,

$$\|b_\lambda(x_1) - b_\lambda(x_2)\|_2 \leqslant (\mathrm{L}_y + \alpha\mathrm{L} + 1/\lambda)\|x_1 - x_2\|_2. \tag{A.10}$$

Let $\bar{\gamma} = (4\lambda)^{-1}(\mathrm{L}_y + \alpha\mathrm{L} + 1/\lambda)^{-2}$. From (A.9) and (A.10), using Lemma SM5.1 in [57] and we get that there exist $\lambda_V \in (0,1]$, $c \geqslant 0$ such that for any $\delta \in (0,\bar{\gamma}]$, R_δ satisfies the discrete drift condition $\mathbf{D}_{\mathrm{d}}\left(V, \lambda_V^\delta, c\delta\right)$.

For any probability measure ν_1, ν_2, from the definition (3.1) and Hölder's inequality we have that

$$\|\nu_1 - \nu_2\|_V \leqslant \|\nu_1 - \nu_2\|_{\mathrm{TV}}^{1/2}\left(\nu_1\left[V^2\right] + \nu_2\left[V^2\right]\right)^{1/2}. \tag{A.11}$$

Since $\bar{\delta} \leqslant \bar{\gamma}$, the contractivity condition (A.8) holds, (A.11) holds, then from Theorem 8 and Corollary 2 in [22], we can find $A_2 \geqslant 0$ and $\rho_2 \in [0,1)$ such that for any $\delta \in (0,\bar{\delta}]$, $x_1, x_2 \in \mathbb{R}^d$, and $k \in \mathbb{N}$,

$$\begin{aligned} \left\|\boldsymbol{\delta}_{x_1}\mathrm{R}_\delta^k - \boldsymbol{\delta}_{x_2}\mathrm{R}_\delta^k\right\|_{\mathrm{TV}} &\leqslant A_2\rho_2^{k\delta}\left(V(x_1) + V(x_2)\right) \\ &\leqslant A_2\rho_2^{k\delta}\left(V^2(x_1) + V^2(x_2)\right) \\ \mathbf{W}_1\left(\boldsymbol{\delta}_{x_1}\mathrm{R}_\delta^k, \boldsymbol{\delta}_{x_2}\mathrm{R}_\delta^k\right) &\leqslant A_2\rho_2^{k\delta}\|x_1 - x_2\|_2. \end{aligned} \tag{A.12}$$

Then we conclude the proof from (A.11). ■

A.7. Proof of theorem 3.12.

Proof. Most of our proof is based on [57] and [22].

Recall that

$$\mathrm{R}_\delta(x,\ \mathrm{A}) = (2\pi)^{-d/2}\int_{\mathbb{R}^d}\mathbf{1}_{\mathrm{A}}\left(x + \delta b_\lambda(x) + \sqrt{2\delta}z\right)\exp\left[-\|z\|^2/2\right]\mathrm{d}z. \tag{A.13}$$

We introduce the stochastic process $\left(\overline{\mathbf{X}}_t\right)_{t\geqslant 0}$, which is exactly the solution of the following SDE:

$$\begin{cases} \mathrm{d}\overline{\mathbf{X}}_t = b_\lambda\left(\overline{\mathbf{X}}_t\right)\mathrm{d}t + \sqrt{2}\,\mathrm{d}\mathbf{B}_t \\ b_\lambda(x) = \nabla\log(p(y|x)) + \alpha\nabla\log q_\theta(x) + \dfrac{\Pi_C(x) - x}{\lambda} \\ \overline{\mathbf{X}}_0 = X_0 \end{cases} \tag{A.14}$$

where $(\mathbf{B}_t)_{t\geqslant 0}$ is a d-dimensional Brownian motion.

From Lemma 3.7, b_λ is $(\mathrm{L}_y + \alpha\mathrm{L} + 1/\lambda)$-Lipschitz continuous. From Chapter 5, Theorem 2.9 of [48] we have that the SDE (A.14) admits a unique strong solution for any initial condition

$\overline{\mathbf{X}}_0$ with $\mathbb{E}\left[\left\|\overline{\mathbf{X}}_0\right\|^2\right] < +\infty$. We denote by $(\mathrm{P}_t)_{t \geqslant 0}$ the semigroup associated with the strong solutions of SDE (A.14). Similarly to the proof of Theorem 3.11, replacing Corollary 2 in [22] by Theorem 21 and Corollary 22 in [22], there exist $\tilde{A}_1 \geqslant 0$ and $\tilde{\rho}_1 \in [0,1)$ such that that for any $x_1, x_2 \in \mathbb{R}^d$ and $t \geqslant 0$,

$$\begin{aligned} \|\boldsymbol{\delta}_{x_1}\mathrm{P}_t - \boldsymbol{\delta}_{x_2}\mathrm{P}_t\|_V &\leqslant \tilde{A}_1\tilde{\rho}_1^t\left(V^2(x_1) + V^2(x_2)\right), \\ \mathbf{W}_1\left(\boldsymbol{\delta}_{x_1}\mathrm{P}_t, \boldsymbol{\delta}_{x_2}\mathrm{P}_t\right) &\leqslant \tilde{A}_1\tilde{\rho}_1^t\,\|x_1 - x_2\|_2\,. \end{aligned} \tag{A.15}$$

Combining (A.15), Theorem 3.11, the fact that $\left(\mathscr{P}_1\left(\mathbb{R}^d\right), \mathbf{W}_1\right)$ is a complete metric space and the Picard fixed point theorem, we can obtain that for any $\delta \in (0, \bar{\delta}]$ there exist $\pi_{\delta,\lambda}, \tilde{\pi}_\lambda \in \mathscr{P}_1\left(\mathbb{R}^d\right)$ such that $\pi_{\delta,\lambda}\mathrm{R}_\delta = \pi_{\delta,\lambda}$ and for any $t \geqslant 0, \tilde{\pi}_\lambda\mathrm{P}_t = \tilde{\pi}_\lambda$. By Theorem 2.1 in [80] we have that for any $x \in \mathbb{R}^d$,

$$(\mathrm{d}\tilde{\pi}_\lambda/\mathrm{dLeb})(x) \propto \exp\left[-\iota_C^{(\lambda)}(x)\right] p(y|x)p_\lambda^\alpha(x), \tag{A.16}$$

Therefore from (2.12) π_λ and $\tilde{\pi}_\lambda$ are exactly the same.

Similar to (3.7), from (A.15) we have that for any $t \geqslant 0$ and $x \in \mathbb{R}^d$,

$$\|\boldsymbol{\delta}_x\mathrm{P}_t - \pi_\lambda\|_V \leqslant \tilde{A}_1\tilde{\rho}_1^t\left(V^2(x) + \int_{\mathbb{R}^d} V^2(\tilde{x})\mathrm{d}\pi_\lambda(\tilde{x})\right) \tag{A.17}$$

Since we already proved that $\int_{\mathbb{R}^d} V^2(\tilde{x})\mathrm{d}\pi_\lambda(\tilde{x}) < +\infty$ in Lemma 3.1, we can find $B_1 \geqslant 0$ such that for any $x \in \mathbb{R}^d$ we have

$$\|\boldsymbol{\delta}_x\mathrm{P}_t - \pi_\lambda\|_V \leqslant B_1\tilde{\rho}_1^t V^2(x). \tag{A.18}$$

Select a large $m_1 \in \mathbb{N}^*$ such that $m_1 \geqslant \bar{\delta}^{-1}$. Let's now consider the interval $[0,l]$, $l \in \mathbb{N}^*$. To compare $\pi_{\delta,\lambda}$ with π_δ, we first construct a continuous time Markov process $X_t^{(1)}$ such that $X_{j/m_1}^{(1)}$ has the same distribution as the j-th sample X_j by NF-ULA (2.13).

Define $b_1\left(t, (w_t)_{t\in[0,l]}\right) = \sum_{j=0}^{m_1 l - 1} \mathbf{1}_{[j/m_1,(j+1)/m_1)}(t) b_\lambda\left(w_{j/m_1}\right)$ and $b_2\left(t, (w_t)_{t\in[0,l]}\right) = b_\lambda(w_t)$. Let $\mathbf{X}_t^{(1)}$ and $\mathbf{X}_t^{(2)}$ be the unique strong solution of SDE $\mathrm{d}\mathbf{X}_t = b\left(t, (\mathbf{X}_t)_{t\in[0,l]}\right)\mathrm{d}t + \sqrt{2}\mathrm{d}\mathbf{B}_t$ with $\mathbf{X}_0 = x \in \mathbb{R}^d$ and $b = b_1$, respectively $b = b_2$. Note that $\left(\mathbf{X}_{k/m_1}^{(1)}\right) = (X_k)_{k\in\mathbb{N}}$ and $\left(\mathbf{X}_t^{(2)}\right)_{t\geqslant 0} = \left(\overline{\mathbf{X}}_t\right)_{t\geqslant 0}$. Denote $P_t^{(1)}$ and $P_t^{(2)}$ the Markov semigroup associated with $\mathbf{X}_t^{(1)}$ and $\mathbf{X}_t^{(2)}$. Then for any $x \in \mathbb{R}^d$, $k \in \mathbb{N}^*$ we have

$$\boldsymbol{\delta}_x\mathrm{R}_{1/m_1}^{km_1} = \boldsymbol{\delta}_x\mathrm{P}_k^{(1)}, \quad \boldsymbol{\delta}_x\mathrm{P}_k = \boldsymbol{\delta}_x\mathrm{P}_k^{(2)}. \tag{A.19}$$

From Lemma 3.7 and A 3.8, for any $t \in [j/m_1, (j+1)/m_1), j \in \{0, \ldots, m_1 l - 1\}$ and $(w_t)_{t\in[0,l]} \in \mathrm{C}\left([0,l], \mathbb{R}^d\right)$ we have that

$$\begin{aligned} \left\|b_1\left(t, (w_t)_{t\in[0,l]}\right) - b_2\left(t, (w_t)_{t\in[0,l]}\right)\right\|^2 &= \left\|b_\lambda\left(w_{j/m_1}\right) - b_\lambda(w_t)\right\|^2 \\ &\leqslant \left(\mathrm{L}_y + \alpha\mathrm{L} + 1/\lambda\right)^2\left\|w_{j/m_1} - w_t\right\|^2. \end{aligned} \tag{A.20}$$

Using Cauchy-Schwarz inequality, Hölder's inequality and Itô's isometry we have for any $t \in [j/m_1, (j+1)/m_1)$,

$$
\begin{aligned}
\mathbb{E}\left[\left\|\mathbf{X}_t^{(2)} - \mathbf{X}_{j/m_1}^{(2)}\right\|^2\right] &= \mathbb{E}\left[\left\|\int_{j/m_1}^t \left(b_\lambda\left(\mathbf{X}_\tau^{(2)}\right) \mathrm{d}\tau + \sqrt{2}\,\mathrm{d}\mathbf{B}_\tau\right)\right\|^2\right] \\
&\leqslant \mathbb{E}\left[2\left\|\int_{j/m_1}^t b_\lambda\left(\mathbf{X}_\tau^{(2)}\right) \mathrm{d}\tau\right\|^2 + 2\left\|\sqrt{2}\left(\mathbf{B}_t - \mathbf{B}_{j/m_1}\right)\right\|^2\right] \\
&\leqslant 2\left(t - \frac{j}{m_1}\right)\mathbb{E}\left[\int_{j/m_1}^t \left\|b_\lambda\left(\mathbf{X}_\tau^{(2)}\right)\right\|^2 \mathrm{d}\tau\right] + 4d\left(t - \frac{j}{m_1}\right) \\
&\leqslant 2\left(t - \frac{j}{m_1}\right)^2 \sup_{\tau \leqslant (j+1)/m_1} \mathbb{E}\left\|b_\lambda\left(\overline{\mathbf{X}}_\tau\right)\right\|^2 + 4d\left(t - \frac{j}{m_1}\right)
\end{aligned}
\tag{A.21}
$$

Since we have proved (A.8), (A.9), (A.10) in Appendix A.6, from Lemma 2.11 and Lemma 2.12 in [65], for any $\tau > 0$ we have

$$
\mathbb{E}\left\|\overline{\mathbf{X}}_\tau\right\|^2 \leqslant B_{0,0}, \tag{A.22}
$$

where $B_{0,0}$ is an upper bound formed by $\lambda, C, b_\lambda(0), d, x$. Then from (A.10) we have that

$$
\mathbb{E}\left\|b_\lambda\left(\overline{\mathbf{X}}_\tau\right)\right\|^2 \leqslant 2\left(\mathrm{L}_y + \alpha\mathrm{L} + 1/\lambda\right)^2 \mathbb{E}\left\|\overline{\mathbf{X}}_\tau\right\|^2 + 2\left\|b_\lambda(0)\right\|^2 \leqslant B_3, \quad \forall \tau > 0, \tag{A.23}
$$

where $B_3 = 2\left(\mathrm{L}_y + \alpha\mathrm{L} + 1/\lambda\right)^2 B_{0,0} + 2\left\|b_\lambda(0)\right\|^2 \geqslant 0$.

Then from (A.20), (A.21), (A.23), for $i \in \{0, \cdots l-1\}$ we have that

$$
\begin{aligned}
&\int_i^{i+1} \mathbb{E}\left[\left\|b_1\left(t, \mathbf{X}_t^{(2)}\right) - b_2\left(t, \mathbf{X}_t^{(2)}\right)\right\|^2\right] \mathrm{d}t \\
&\leqslant \sum_{j=im_1}^{(i+1)m_1-1} \int_{j/m_1}^{(j+1)/m_1} \mathbb{E}\left[\left\|b_1\left(t, \mathbf{X}_t^{(2)}\right) - b_2\left(t, \mathbf{X}_t^{(2)}\right)\right\|^2\right] \mathrm{d}t \\
&\leqslant m_1(\mathrm{L}_y + \alpha\mathrm{L} + 1/\lambda)^2 \int_{j/m_1}^{(j+1)/m_1} \mathbb{E}\left[\left\|\mathbf{X}_t^{(2)} - \mathbf{X}_{j/m_1}^{(2)}\right\|^2\right] \mathrm{d}t \\
&\leqslant (\mathrm{L}_y + \alpha\mathrm{L} + 1/\lambda)^2 \left(\frac{2B_3}{3m_1^2} + \frac{2d}{m_1}\right).
\end{aligned}
\tag{A.24}
$$

From (A.19) and Lemma SM6.1 in [57], we obtain that there exists $B_b \geqslant 0$ such that for

any $x \in \mathbb{R}^d$,

(A.25)
$$\begin{aligned}
&\left\|\boldsymbol{\delta}_x \mathrm{R}_{1/m_1}^{lm_1} - \boldsymbol{\delta}_x \mathrm{P}_l\right\|_V = \left\|\boldsymbol{\delta}_x \mathrm{P}_l^{(1)} - \boldsymbol{\delta}_x \mathrm{P}_l^{(2)}\right\|_V = \left\|\boldsymbol{\delta}_x \mathrm{P}_l^{(2)} - \boldsymbol{\delta}_x \mathrm{P}_l^{(1)}\right\|_V \\
&\leqslant \left(\boldsymbol{\delta}_x \mathrm{P}_l^{(1)}\left[V^2\right] + \boldsymbol{\delta}_x \mathrm{P}_l^{(2)}\left[V^2\right]\right)^{1/2} \times \left(\sum_{i=0}^{l-1} \int_i^{i+1} \mathbb{E}\left[\left\|b_1\left(t, \mathbf{X}_t^{(2)}\right) - b_2\left(t, \mathbf{X}_t^{(2)}\right)\right\|^2\right] \mathrm{d}t\right)^{1/2} \\
&\leqslant (\mathrm{L}_y + \alpha \mathrm{L} + 1/\lambda)\sqrt{l\left(\frac{2B_3}{3m_1^2} + \frac{2d}{m_1}\right)}\left(\boldsymbol{\delta}_x \mathrm{P}_t^{(1)}\left[V^2\right] + \boldsymbol{\delta}_x \mathrm{P}_t^{(2)}\left[V^2\right]\right)^{1/2}.
\end{aligned}$$

Assume that there is a function $W \in \mathrm{C}^2\left(\mathbb{R}^d, [1, +\infty)\right)$ such that $\lim_{\|x\| \to +\infty} W(x) = +\infty$. Recall that from (A.9), using Lemma SM5.1 in [57] and we get that there exist $\lambda_W \in (0, 1]$, $c, \beta \geqslant 0$ and $\zeta > 0$ such that for any $\delta \in \left(0, (4\lambda)^{-1}\left(\mathrm{L}_y + \alpha \mathrm{L} + 1/\lambda\right)^{-2}\right]$, R_δ satisfies the discrete drift condition $\mathbf{D}_{\mathrm{d}}\left(W, \lambda_W^\delta, c\delta\right)$ and $(\mathrm{P}_t)_{t \geqslant 0}$ satisfies the continuous drift condition $\mathbf{D}_{\mathrm{c}}(W, \zeta, \beta)$. From Lemma SM5.2 in [57], there exists $B_c \geqslant 0$ such that for any $x \in \mathbb{R}^d, t \geqslant 0$ and $k \in \mathbb{N}^*$ we have

$$\mathrm{R}_\delta^k W(x) + \mathrm{P}_t W(x) \leqslant B_c^2 W(x). \tag{A.26}$$

Let $W(x) = V^2(x)$ and $k = m_1 l$, $\delta = 1/m_1$, $t = l$, then $\forall x \in \mathbb{R}^d$,

$$\boldsymbol{\delta}_x \mathrm{P}_l^{(1)}\left[V^2\right] + \boldsymbol{\delta}_x \mathrm{P}_l^{(2)}\left[V^2\right] \leqslant B_c^2 V^2(x) \tag{A.27}$$

Combined with (A.25), we have that

$$\left\|\boldsymbol{\delta}_x \mathrm{R}_{1/m_1}^{m_1 l} - \boldsymbol{\delta}_x \mathrm{P}_l\right\|_V \leqslant B_c V(x)(\mathrm{L}_y + \alpha \mathrm{L} + 1/\lambda)\sqrt{l\left(\frac{2B_3}{3m_1^2} + \frac{2d}{m_1}\right)} \tag{A.28}$$

To give a bound on $\left\|\boldsymbol{\delta}_x \mathrm{R}_{1/m_1}^{m_1 l} - \pi_\lambda\right\|_V$, we use triangular inequality to split it into two terms:

$$\left\|\boldsymbol{\delta}_x \mathrm{R}_{1/m_1}^{m_1 l} - \pi_\lambda\right\|_V \leqslant \left\|\boldsymbol{\delta}_x \mathrm{R}_{1/m_1}^{m_1 l} - \boldsymbol{\delta}_x \mathrm{P}_l\right\|_V + \left\|\boldsymbol{\delta}_x \mathrm{P}_l - \pi_\lambda\right\|_V. \tag{A.29}$$

Using this result and (A.18), we obtain that there exists $B_1, B_2 \geqslant 0$ such that for any $m_1 \in \mathbb{N}^*$ with $1/m_1 \leqslant \bar{\delta}$,

$$\left\|\boldsymbol{\delta}_x \mathrm{R}_{1/m_1}^{m_1 l} - \pi_\lambda\right\|_V \leqslant B_1 \tilde{\rho}_1^l V^2(x) + B_2 V(x)\sqrt{l\left(\frac{B_3}{3m_1^2} + \frac{d}{m_1}\right)}. \tag{A.30}$$

The proof in the general case where $\delta \in (0, \bar{\delta}]$ is similar when the interval $[0, l]$ is changed to $[0, lm_1\delta]$.

Then we obtain that there exists $B_1, B_2, B_3 \geqslant 0$, $\tilde{\rho}_1 \in [0, 1)$ such that for any $\delta \in (0, \bar{\delta}]$, $k \in \mathbb{N}^*$,

$$\left\|\boldsymbol{\delta}_x \mathrm{R}_\delta^k - \pi_\lambda\right\|_V \leqslant B_1 \tilde{\rho}_1^{k\delta} V^2(x) + B_2 V(x)\sqrt{\delta^2 k\left(d + \frac{B_3\delta}{3}\right)}. \tag{A.31}$$

■

Appendix B. Additional experiments.

The second limited-angle computed tomography reconstruction experiment we test is using the Poisson noise, where the model can be formulated as $y \sim P(Ax)$ and $P(Ax)$ denotes adding a Poisson noise on Ax. We simulate the noisy sinogram as

$$y = -\frac{1}{\mu} \log \left(\frac{N_1}{N_0} \right), \quad N_1 \sim \text{Poisson} \left(N_0 \exp(-A(x)\mu) \right).$$

Here $N_0 = 4096$ is the mean photon count per detector bin without attenuation. $\mu = 0.05$ is a constant. Since Poisson noise implies a different likelihood

$$p(y|x) = \frac{1}{K_0} \exp(-J(x, y)),$$
$$J(x, y) = \sum_{i=1}^{m} e^{-A(x)_i \mu} N_0 + e^{-y_i \mu} N_0 \left(A(x)_i \mu - \log \left(N_0 \right) \right),$$

we calculate $\nabla \log p(y|x) = -\nabla J(x, y)$ by using the auto-gradient library.

We select a different $\alpha = 4000$ for NF-ULA while keeping all the other settings the same as in the main paper.

Both PnP-ULA and NF-ULA have burn-in iterations of more than 20000. After the burn-in time, we calculate the posterior mean and the standard deviation by obtaining 10000 samples and computing the PSNR of the samples' mean. For Poisson noise, the likelihood is more complicated than Gaussian, and NF-ULA spends 510s.

Fig 8 includes the original image, the FBP, the posterior mean and the standard deviation of PnP-ULA (realSN-DnCNN) and NF-ULA (patchNR). Table 5 provides the PSNR of the posterior mean. All the samples generated in Table 5 never escape $[-0.2, 1.2]^d$, indicating that the projection $\Pi_C(x)$ is never activated. Note that the huge uncertainties of standard deviation on the left area in the Gaussian-noise case in the main paper are slightly alleviated in the Poison noise experiments. The ACF test results are similar to the CT experiment with Gaussian noise, therefore here we do not repeat them again.

Figure 8. *Limited-view CT reconstruction with Poisson noise. Column 1: Original image. Column 2: Filtered back projection (FBP). Columns 3, and 4: Posterior mean and the standard deviation of the samples generated by PnP-ULA (realSN-DnCNN). Columns 5, and 6: Posterior mean and the standard deviation of the samples generated by NF-ULA (patchNR). PSNR values of the sample mean images are provided in Table 5.*

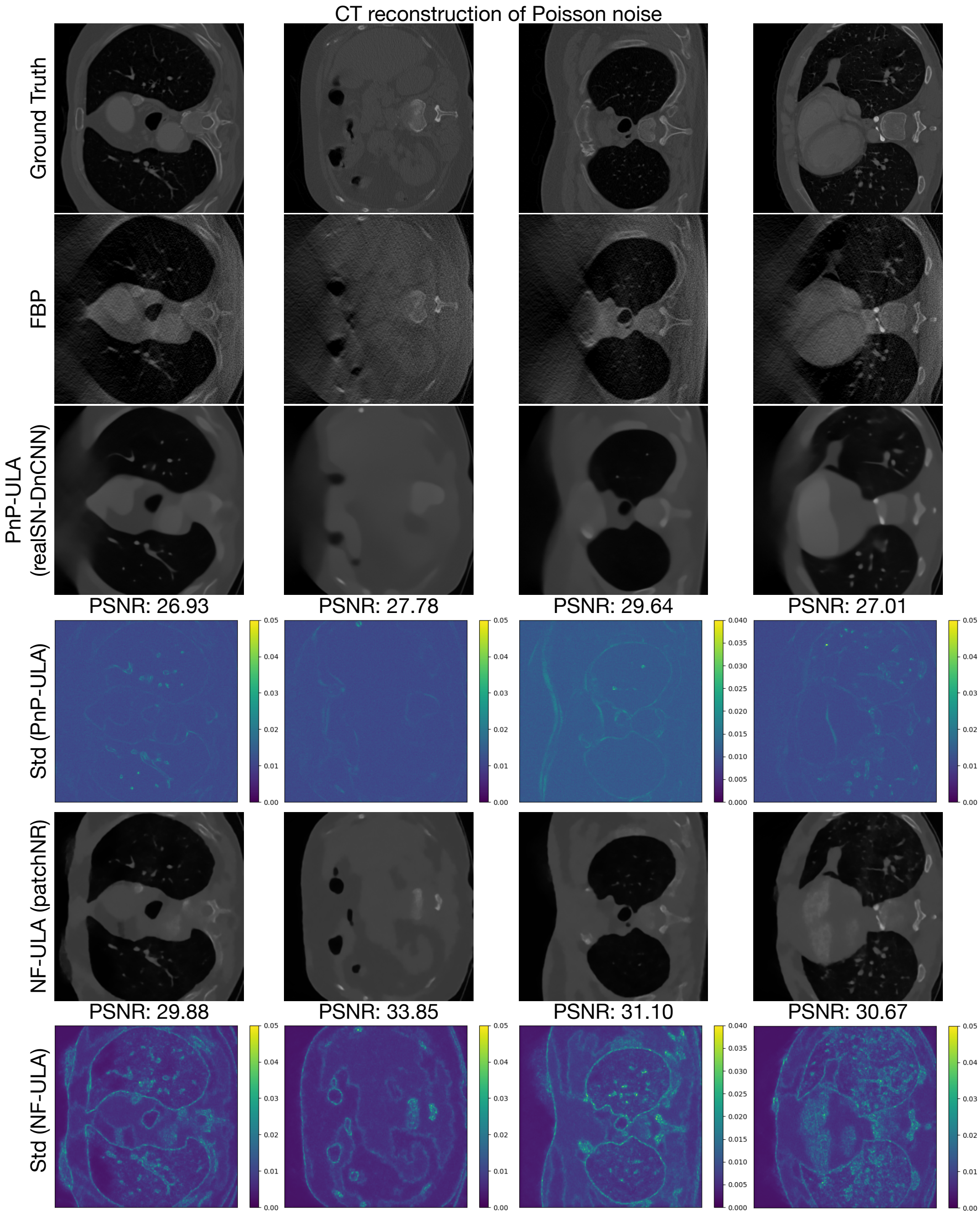

Table 5
CT reconstruction of Poisson noise, limited angles.

CT	$C = [-100, 100]^d$		
	network	parameters	PSNR
figure1			
NF-ULA	PatchNR	$\alpha = 4000$	29.88
PnP-ULA	realSN-DnCNN	$\alpha = 3$	26.93
figure2			
NF-ULA	PatchNR	$\alpha = 4000$	33.85
PnP-ULA	realSN-DnCNN	$\alpha = 3$	27.78
figure3			
NF-ULA	PatchNR	$\alpha = 4000$	31.10
PnP-ULA	realSN-DnCNN	$\alpha = 3$	29.64
figure4			
NF-ULA	PatchNR	$\alpha = 4000$	30.67
PnP-ULA	realSN-DnCNN	$\alpha = 3$	27.01
